# Transcription elongation factor Brd4-P-TEFb accelerates intestinal differentiation-associated *SLC2A5* gene expression

**DOI:** 10.1016/j.bbrep.2016.05.016

**Published:** 2016-06-01

**Authors:** Yuko Inamochi, Anup Dey, Akira Nishiyama, Takeo Kubota, Keiko Ozato, Toshinao Goda, Kazuki Mochizuki

**Affiliations:** a Laboratory of Nutritional Physiology, Graduate School of Nutritional and Environmental Sciences and Global COE, Laboratory of Nutritional Physiology, University of Shizuoka, Shizuoka, Japan; b Laboratory of Molecular Growth Regulation, NICHD, NIH, Bethesda, MD, USA; c Division of Engineering, Interdisciplinary Graduate School of Medicine and Engineering, University of Yamanashi, Chuo, Yamanashi, Japan; d Laboratory of Food and Nutritional Sciences, Department of Local Produce and Food Sciences, Faculty of Life and Environmental Sciences, University of Yamanashi, Yamanashi, Japan

**Keywords:** Histone acetylation, Fructose transporter, P44/42 mapk, Glucocorticoid hormone, Small intestine

## Abstract

**Background:**

Expression of the fructose transporter gene *SLC2A5* and histone acetylation in the transcribed region are induced by differentiation associated-signals such as glucocorticoids and p44/42 mitogen-activated protein kinase (MAPK) inhibition in small intestinal Caco-2 cells.

**Methods:**

We co-treated with glucocorticoid receptor agonist dexamethasone (Dex) and p44/42 MAPK inhibitor PD98059 (PD) in Caco-2 cells with or without Brd4 small hairpin (sh) RNA expression vector, and the cells were analyzed by qRT-PCR and chromatin immunoprecipitation assays. The small intestine of wild-type mice and *Brd4*^+/−^ mice during weaning period were analyzed by qRT-PCR.

**Results:**

Co-treatment with Dex and PD increased binding of the bromodomain-containing protein-4 (Brd4)–positive transcriptional elongation factor-b (P-TEFb)–RNA polymerase II complex to acetylated histones in the transcribed region of *SLC2A5*. Brd4-protein depletion by shRNA revealed that the association of these proteins on the transcribed region of *SLC2A5* promoted gene expression in a Brd4-dependent manner. Expression of small-intestine *Slc2a5*, but not another intestinal gene sucrase-isomaltase, during weaning period, was significantly lower in *Brd4*^+/−^ mice compared with wild-type mice.

**Conclusions:**

Brd4-P-TEFb plays a crucial role in differentiation-associated transcription of *SLC2A5* gene in intestinal Caco-2 cells and in the small intestine of mice during weaning period.

**General significance:**

Histone acetylation and the transcription elongation factor Brd4 are important for *SLC2A5* expression in the small intestine.

## Highlights

• Brd4 regulates expression of *SLC2A5* in human intestinal Caco-2 cells.
• Brd4–P-TEFb and acetylated histones bind transcribed region of *SLC2A5.*
• Brd4 regulates jejunal *Slc2a5* expression during weaning period of mice.

## 1 Introduction

Stem cells in the crypt of the small intestine rapidly differentiates to absorptive cells in the villus, and start to express many genes related digestion and absorption of nutrients. Indeed, previous studies, including our own, have shown that the expression of the hexose transporter, *SLC2A5*, generally called as glucose transporter 5 (GLUT5), which is involved in fructose absorption from the lumen, is induced during the transition from the crypt to the villus [1]. It is known that glucocorticoids and inactivation of p44/42 mitogen-activated protein kinase (MAPK), also known as extracellular signal-regulated kinase (ERK)1/2, play important roles in intestinal differentiation and gene expression [2]. Expression of the *Slc2a5* gene was increased when serum concentrations of corticosterone were elevated during weaning in rats [3]. Injection of the glucocorticoid receptor agonist dexamethasone (Dex) into suckling rats induced the expression of small-intestine *Slc2a5* [4]. p44/42 MAPK was inactivated prior to differentiation of the fetal small intestine in rats and in human intestinal Caco-2 cells [5]. Furthermore, endogenous levels of epidermal growth factor, which activates p44/42 MAPK, were decreased in the rat small intestine during weaning, because it is provided via the mother's milk [3]. Inhibition of p44/42 MAPK was recently shown to enhance glucocorticoid-mediated *SLC2A5* expression in human intestinal Caco-2 cells [6]. These evidences suggest that glucocorticoids and p44/42 MAPK inactivation could coordinately enhance *SLC2A5* expression in small-intestine cells during the differentiation.

Previous studies showed that hormone-induced gene expression, which frequently occurs in differentiating cells, is mediated by epigenetic memories which are acquired modifications on the chromatin such as modifications of the histone tail, including acetylation, methylation and phosphorylation, and the DNA methylation [7]. Acetylation of histones H3 and H4 is associated with the euchromatin region and transactivation [8]. We previously demonstrated that co-treatment of Caco-2 cells with Dex and PD98059 (PD), which inhibits p44/42 MAPK activation, enhanced the acetylation of histones H3 and H4 around *SLC2A5,* particularly in the transcribed region of the gene [9]. These results suggest that, under these conditions, induction of *SLC2A5* expression is regulated not only by activation of glucocorticoid receptors (GRs), but also by enhancing the histone acetylation on *SLC2A5*.

Acetylation of histones H3 and H4 in the promoter region of genes facilitates binding of the transcriptional machinery, including transcription factors, co-activators, the SWItch/Sucrose NonFermentable (SWI/SNF) complex, and RNA polymerase II [10]. However, the significance of histone acetylation in transcribed regions remains poorly understood. Bromodomain-containing protein-4 (Brd4), which binds acetylated histones, was recently shown to enhance gene expression by recruiting the mRNA transcription elongation complex to acetylated histones in transcribed regions of genes [11], [12]. This complex, known as positive transcription elongation factor-b (P-TEFb), is a heterodimer of cyclin T1–Cdk9. These results indicate that Brd4 regulates transcription elongation by recruiting P-TEFb to the transcribed region of genes. Brd4 is the only bromodomain-containing protein known to recruit P-TEFb to acetylated histones in transcribed regions. Brd4 is also highly expressed in the small intestine [13], and histone acetylation in the transcribed region of *SLC2A5* was enhanced by co-treatment with PD and Dex in Caco-2 cells [9]. However, whether functional genes, including *SLC2A5*, are regulated by Brd4-P–TEFb in differentiated cells has not yet been elucidated.

In this study, we examined the possible function of Brd4 downstream of glucocorticoids and p44/42 MAPK inhibition in regulating *SLC2A5* expression in Caco-2 cells. We also investigated the role of Brd4 in *SLC2A5* induction in the small intestine during the suckling–weaning transition using Brd4 heterogeneous gene targeting in mice. Our results in current study suggest that epigenetic regulation via histone acetylation and the Brd4 play vital roles in induction of *SLC2A5* expression during the intestinal differentiation.

## 2 Materials and methods

### 2.1 Cell culture

Caco-2 cells (American Type Culture Collection, Rockville, MD, USA) were seeded at a density of 0.6×10^4^ cells/cm^2^ in 10-cm culture collagen plates (Iwaki, Tokyo, Japan) in Dulbecco's Modified Eagle's Medium (DMEM) containing 10% fetal calf serum (FCS), 1% non-essential amino acids (Invitrogen, Carlsbad, CA, USA), 20 mM HEPES (pH 7.4), 1× antibiotic–antimycotic mixed stock solution (Nakaraitesk, Kyoto, Japan), and 2 mM l-glutamate (Invitrogen) at 37 °C in a humidified atmosphere of 5% CO_2_.

Control short hairpin (sh) RNA- or Brd4-shRNA-expressing Caco-2 cells were constructed by inserting control or Brd4 shRNA into the pSUPERRNAi vector (Oligoengine, Seattle, WA, USA) [14]. The Brd4 shRNA sequence was: 5′-GATCCCCGAAAAGAGGAAGTGGAAGAGATTCAAGAGATCTCTTCCACTTCCTCTTTCTTTTTA-3′, and the control shRNA sequence was: 5′-GATCCCCATGCACGTGCACATATCCCTTCAAGAGAGGGATATGTGCACGTGCATTTTTTGGAAA-3′. These constructs were separately transfected with the plasmid vectors pGag-pol and pAmpho into HEK293T packaging cells, and the supernatants were collected as virus-containing medium 2 days after transfection. Cells were transfected with virus-containing medium mixed with 6 μg polybrene by centrifugation (1000*g*) for 2 h. Transfected cells were selected with puromycin (Sigma Aldrich, St. Louis, MO, USA) for 7 days and cultured in the same conditions described above. After 2 days, cells had reached approximately 70% confluence and normal Caco-2 and control/Brd4-shRNA-expressing cells were continuously cultured in DMEM containing 10% FCS stripped of glucocorticoids by treatment with charcoal/dextran, with 1 µM Dex (glucocorticoid receptor agonist) and 50 µM PD (p44/42 MAPK kinase inhibitor), or vehicle (DMSO) alone for 48 h.

### 2.2 Targeted disruption of *Brd4* gene in mice

*Brd4* heterozygous allele (*Brd4*^+/−^) mice were generated by our group in an NIH animal facility. The mice were derived from C57BL/6J mice and bred with wild-type mice purchased from Japan Oriental Yeast Co. Ltd. (Shizuoka, Japan). *Brd4*^+/−^ mice were divided into four groups analyzed at the following times after birth: 10 days (n=20 mice; control, 10; *Brd4*^+/–^, 10), 14 days (n=16 mice; control, 11; *Brd4*^+/–^, 5), 21 days (n=14 mice; control, 7; *Brd4*^+/–^, 7), and 26 days (n=17 mice; control, 9; *Brd4*^+/–^, 8). Animals were genotyped by polymerase chain reaction (PCR) using the following primers: WT forward: 5′-GGACTAGAAACCTCCCAAATGTCTACAA-3′; neo forward: 5′-TGAAGAGCTTGGCGGCGAATGGG-3′; both reverse: 5′-CCTGTGTGCACTTGCTCCCGAGGAGAGA-3′. The PCR conditions for WT were 94 °C for 1 min, and 34 cycles of 94 °C for 20 s, 60 °C for 20 s, and 72 °C for 2 min, and 72 °C for 7 min. The real-time reverse transcription (RT)-PCR conditions for neo were 95 °C for 5 min, and 30 cycles of 95 °C for 10 s, 60 °C for 10 s, and 72 °C for 1 min 30 s, and 95 °C for 10 s, 65 °C for 15 s, and 50 °C for 10 s. Mice were maintained in an air-conditioned room at 23±2 °C and a humidity of 55±5% with a 12-h light–dark cycle (7:00–19:00) at the Japan Oriental Yeast Co. Ltd. Mice were killed by decapitation between 12:00 and 15:00. All experimental procedures were approved by the Institutional Animal Care and Use Committee of the University of Shizuoka.

### 2.3 RNA analysis

Total RNA was subjected to RT using Superscript III reverse transcriptase (Invitrogen). Test genes (Caco-2 cells: *SLC2A5*; mice: *Slc2a5, Si*, and *Brd4*) and housekeeping genes (Caco-2 cells: β-actin [*ACTB*], mice: 16 S rRNA) were amplified by real-time RT-PCR using a Light Cycler System (Roche Molecular Biochemicals, Bavaria, Germany) and SYBR Green I (Takara Bio, Shiga, Japan). The cycle threshold (CT) values detected by real-time RT-PCR were converted into signal intensities using the delta-delta method [15]. The sequences of the primers for real-time RT-PCR are listed in Supplemental Table 1.

### 2.4 Immunoblotting

Total cell proteins were extracted in RIPA buffer (1% Nonidet P-40, 0.1% sodium dodecyl sulfate [SDS], 20 mM Tris–HCl pH 8.0, 5 mM ethylenediamine tetraacetic acid, 150 mM NaCl, protease inhibitor tablet (Complete Mini, Roche Molecular Biochemicals)/10 ml, and phosphatase inhibitors [1 mM NaMoO_4_, 50 mM NaF, and 1 mM Na_3_VO_4_]). Lysates were centrifuged at 10,000*g* for 10 min at 4 °C. The protein concentration of the soluble supernatants was determined by the Lowry method, and samples were stored at −20 °C.

**Fig. 1:**
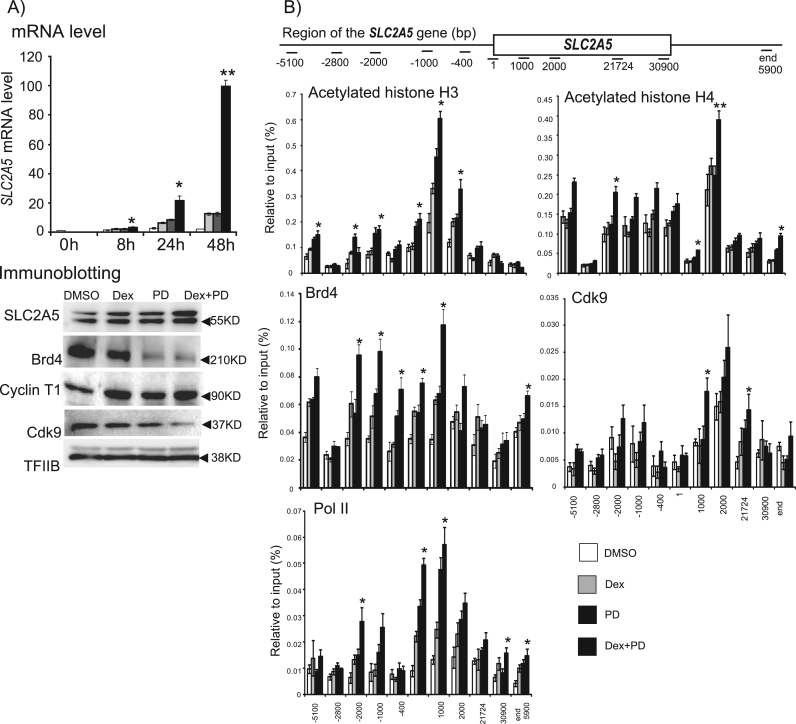
Expression of *SLC2A5* in Caco-2 cells treated with Dex and/or PD. (A) *SLC2A5* mRNA in cells treated with Dex and/or PD for 8, 24, or 48 h, and protein levels of SLC2A5, Brd4, Cyclin T1, Cdk9, and TFIIB in cells treated with Dex and/or PD for 48 h. (B) ChIP assays for acetylated histone H3 at K9/14, acetylated histone H4 at K5/8/12/16, Brd4, Cdk9, and Pol II around the *SLC2A5* gene in cells treated with Dex and/or PD for 48 h. Means±SEM of six (RNA) or five (ChIP assays) experiments are shown. *P <0.05 and **P <0.01 compared with control cells (DMSO).

**Fig. 2:**
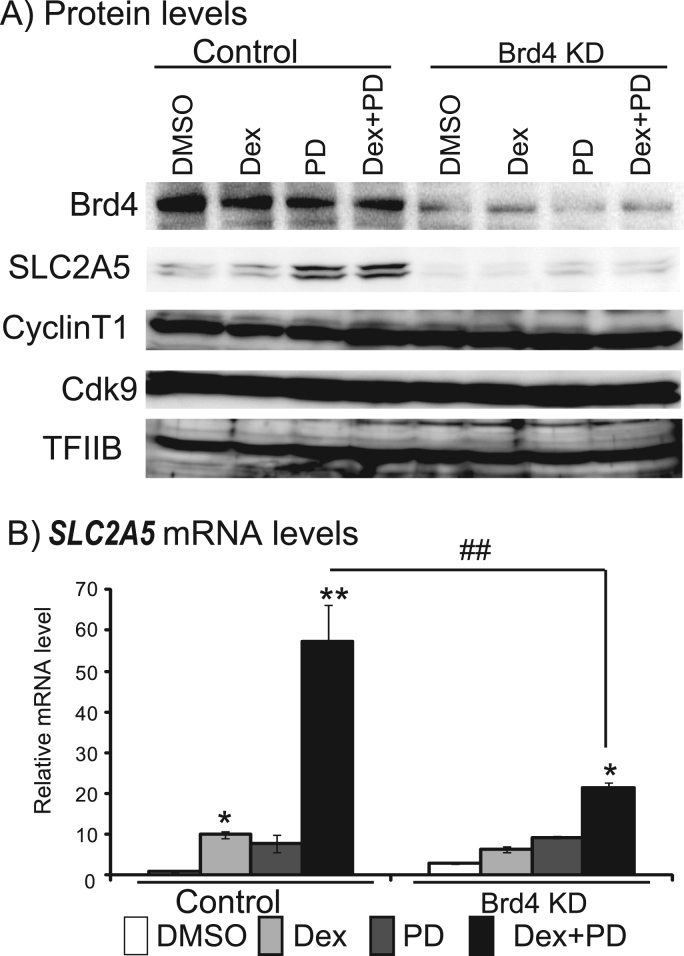
*SLC2A5* mRNA expression in Dex and/or PD-treated Brd4-depleted cells. Protein levels of SLC2A5, Brd4, Cyclin T1, Cdk9, and TFIIB (A) and *SLC2A5* mRNA (B) in cells treated with Dex and/or PD for 48 h. Means±SEM of six mRNA determinations are shown. ^*^P <0.05 and ^**^P <0.01 compared with control cells (DMSO). ^##^P <0.01 compared with control cells.

Total proteins (60 μg, Fig. 1A; 70 μg, Fig. 2A) were separated by 10% SDS–polyacrylamide gel electrophoresis and transferred to Immobilon membranes (Millipore; Billerica, MA, USA) at 80 V for 120 min in Tris/glycine/methanol transfer buffer. The membranes were blocked for 30 min in 3% skim milk in phosphate-buffered saline (PBS) with 0.05% Tween 20, pH 7.4 (PBS–Tween) at room temperature. The membranes were then incubated in 3% skim milk in PBS–Tween at 4 °C for >7 h with primary antibodies against Brd4 [16], cyclin T1 (Abcam, Cambridge, MA, USA), Cdk9 (Santa Cruz Biotechnology; Santa Cruz, CA, USA), and TFIIB (Santa Cruz Biotechnology). After washing in PBS–Tween, the membranes were incubated with biotin-conjugated anti-rabbit IgG (GE Healthcare, Little Chalfont, UK) in 3% PBS–Tween. The membranes were then washed in PBS–Tween and incubated with horseradish peroxidase-conjugated anti-biotin third antibody (Cell Signaling Technology, MA, USA). Signals were detected by chemiluminescence (ECL Plus, GE Healthcare), according to the manufacturer's instructions.

### 2.5 Chromatin immunoprecipitation (ChIP) assay

Cells were incubated in fixation solution (1% formaldehyde, 4.5 mM HEPES pH 8.0, 9 mM NaCl, 0.09 mM EDTA, 0.04 mM ethylene glycol tetraacetic acid) in 10% FCS/DMEM for 30 min at 37 °C. Reactions were terminated by adding glycine to a final concentration of 150 mM. After washing in fluorescence-activated cell sorting solution (1× PBS, 2% bovine serum, 0.05% NaN_3_), the samples were sonicated in SDS lysis buffer (50 mM Tris–HCl pH 8.0, 10 mM EDTA pH 8.0, 1% SDS, 0.5 mM phenylmethanesulfonylfluoride) to generate DNA fragments of 200–500 bp. The ChIP assay was performed as described previously [16], using 1 μg antibodies against acetyl histone H3 at K9/14 (Millipore), acetyl histone H4 at K5/8/12/16 (Millipore), Brd4 (custom antibody generated by the Sigma Custom Antibody service using the Brd4C-terminal peptide CFQSDLLSIFEENLF), Cdk9 (Santa Cruz Biotechnology), RNA polymerase II (Pol II) (Covance, Princeton, NJ, USA), or normal rabbit IgG. The precipitated DNA was subjected to real-time PCR using primers corresponding to the indicated sites in the promoter/enhancer and transcribed regions. The CT values of the ChIP signals detected by real-time PCR were converted to percentages of the signal for the input DNA using the delta-delta method [15], with the formula 100×[2^(CT input–CT IP sample)^]. The primer sequences used in ChIP assays are listed in Supplemental Table 1.

### 2.6 Statistical analysis

**Fig. 3:**
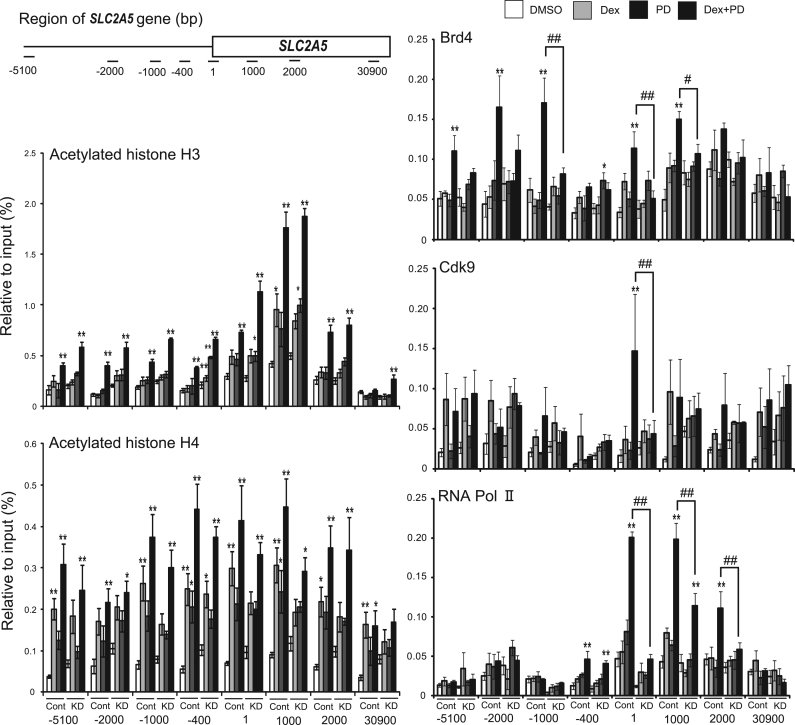
Binding of acetylated histone- and chromatin-remodeling factors around *SLC2A5* in Brd4-depleted cells. ChIP assays for acetylated histone H3 at K9/14, acetylated histone H4 at K5/8/12/16, Brd4, Cdk9, and Pol II around the *SLC2A5* gene in control-shRNA- and Brd4-shRNA-expressing cells treated with Dex and/or PD for 48 h. Means±SEM of five experiments are shown. ^*^P <0.05 and ^**^P <0.01 compared with control cells (DMSO). ^#^P <0.05 and ^##^P <0.01 compared with control cells.

**Fig. 4:**
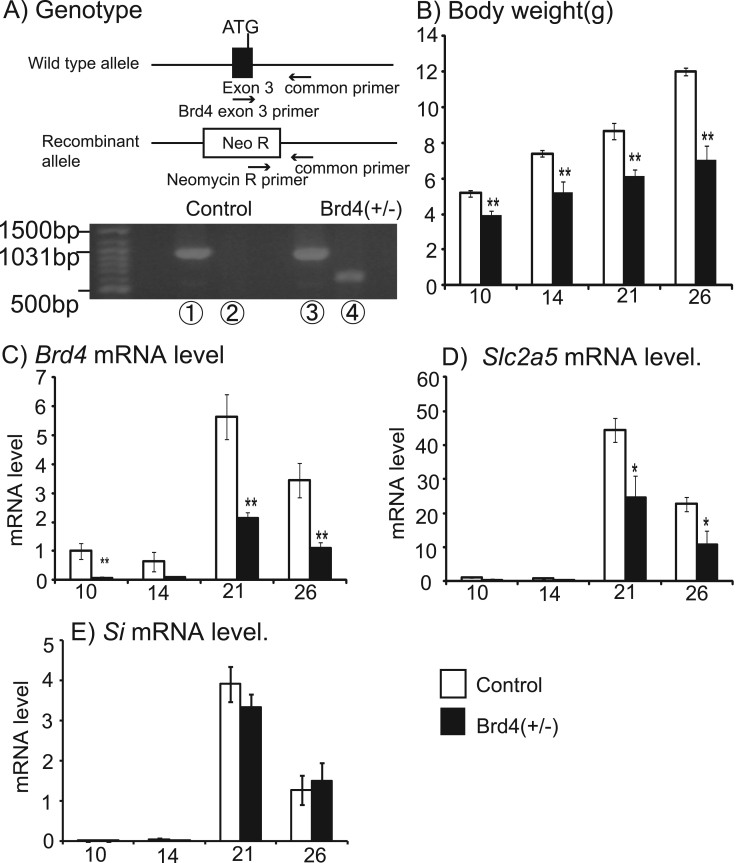
Effects of Brd4 deficiency on mouse small-intestine *SLC2A5* expression during the suckling–weaning period. (A) Mice were genotyped by PCR using DNA isolated from the tail. Body weights (B), mRNA levels for *Brd4* (C), *Slc2a5* (D), and *Si* (E) in control and *Brd4*^+/−^ mice at 10, 14, 21, and 26 days after birth. Numbers of X-axis in (B)–(E) indicate days after birth of mice. Means±SEM for between five and 11 samples are shown. ^*^P <0.05 and ^**^P <0.01 compared with control mice.

Results were expressed as mean±standard error of the mean (SEM). The significance of differences among groups was determined by Tukey's multiple range test based on one-way analysis of variance (ANOVA) (Fig. 1), two-way ANOVA (Fig. 2, Fig. 3), or Student's *t*-test (Fig. 4). A P value <0.05 was considered statistically significant.

## 3 Results

### 3.1 Co-treatment with Dex and PD enhances *SLC2A5* mRNA and binding of acetylated histones and Brd4–P-TEFb–Pol II around the SLC2A5 gene in Caco-2 cells

We performed real-time RT-PCR and ChIP assays in Caco-2 cells treated with 1 µM Dex, an agonist for glucocorticoid receptor, with or without 50 µM PD, a p44/42 MAPK inhibitor. *SLC2A5* expression was significantly increased by co-treatment with Dex and PD for 8, 24, and 48 h, and SLC2A5 protein levels were significantly elevated by co-treatment with Dex and PD for 48 h. Brd4, Cyclin T1, Cdk9, and TFIIB were expressed in Caco-2 cells treated with DMSO, Dex, PD, or Dex and PD. However, Brd4 protein levels were decreased by PD or co-treatment with Dex plus PD (Fig. 1A).

All ChIP signals for IgG were <0.02% relative to input signals. ChIP signals from upstream/transcribed regions associated with acetylated H3 (K9/14) were markedly enhanced by co-treatment with Dex and PD for 48 h, particularly the transcribed regions of *SLC2A5* at −5100 bp and from −1000 bp to 2000 bp. Signals throughout the upstream/transcribed region of *SLC2A5* associated with acetylated histone H4 (K5/8/12/16) were slightly enhanced by co-treatment with Dex and PD for 48 h, and signals from 2000 bp of *SLC2A5* were significantly higher. Co-treatment also notably increased the ChIP signals associated with Brd4 on the promoter/enhancer region and the transcribed region close to the initiation site of *SLC2A5* from −2000 bp to 1000 bp. Co-treatment with Dex and PD for 48 h strongly increased the ChIP signals associated with Cdk9 on the transcribed region close to the initiation site of *SLC2A5* at 1000 bp and 22,000 bp. ChIP signals were also significantly increased in association with Pol II on the promoter/enhancer region and the transcribed region close to the initiation site of *SLC2A5* at −2000 bp, 1 bp, and 1000 bp (Fig. 1B).

### 3.2 Effects of Brd4 depletion on *SLC2A5* expression and binding of acetylated histones and Brd4–P-TEFb–Pol II around the *SLC2A5* gene in Caco-2 cells co-treated with Dex and PD

We generated Caco-2 cell lines stably expressing Brd4 shRNA to determine if Brd4 was necessary for the induction of *SLC2A5* in Caco-2 cells co-treated with Dex and PD. Brd4 protein levels were reduced in these cells compared with controls. Levels of Cyclin T1, Cdk9, and TFIIB proteins were similar in cells subjected to different treatments in both control- and Brd4-shRNA-expressing cells. In contrast, co-treatment with Dex and PD for 48 h increased SLC2A5 levels in control cells, but not in Brd4-shRNA-expressing cells (Fig. 2A). We obtained similar results for *SLC2A5* mRNA levels, with a significant difference between co-treated control- and Brd4-shRNA-expressing cells (Fig. 2B).

Acetylation of histone H3 K9/14 on the promoter/enhancer and transcribed regions of *SLC2A5* (control: from −5100 bp to 2000 bp; Brd4 shRNA: from −5100 bp to 30,900 bp) increased after co-treatment with Dex and PD for 48 h in both control- and Brd4-shRNA-expressing cells (Fig. 4B). Acetylation of histone H4 K5/8/12/16 on the promoter/enhancer and transcribed regions of *SLC2A5* (control: from −5100 bp to 30,900 bp; Brd4 shRNA: from −5100 bp to 2000 bp) also increased after co-treatment with Dex and PD in both cell lines. We obtained similar results in terms of the associations of Brd4, Cdk9, and Pol II with *SLC2A5* in control cells and in WT cells in Fig. 1. However, this association was significantly reduced in Brd4-depleted cells (Fig. 3).

### 3.3 Effects of Brd4 depletion on small-intestine *Slc2a5* gene expression during postnatal development in mice

We determined if Brd4 regulated the expression of *Slc2a5* in the mouse small intestine by real-time RT-PCR using total RNA extracted from WT or *Brd4*^+/−^ mice at 10, 14, 21, and 26 days after birth. The genetic background of *Brd4* heterozygous (*Brd4*^+/−^) mice is shown in Fig. 4. This covered the suckling–weaning transition period when serum glucocorticoid levels increase and p44/42 MAPK is inactivated in rodents. Body weight was lower in *Brd4*^+/−^ mice compared with WT mice at each day after birth. We confirmed that *Brd4* expression was significantly lower in *Brd4*^+/−^ mice compared with WT mice. *Slc2a5,* but not *Si* expression was also significantly lower in *Brd4*^+/−^ mice compared with WT mice at 21 and 26 days after birth (Fig. 4.).

## 4 Discussion

In this study, we demonstrated that co-treatment with Dex and PD induced *SLC2A5* expression as well as acetylation of histones around the *SLC2A5* gene in Caco-2 cells. Furthermore, co-treatment with Dex and PD also promoted the association of Brd4 around *SLC2A5*. Notably, although Brd4 protein levels were reduced by PD and co-treatment with PD and Dex, Brd4 binding around *SLC2A5* was strongly enhanced by co-treatment with PD and Dex. Furthermore, we demonstrated that co-treatment with Dex and PD promoted the association of P-TEFb and Pol II on the transcribed region close to the transcription initiation site of *SLC2A5* in Caco-2 cells. These results were confirmed by RNA interference, which showed that these associations and the expression of *SLC2A5* were Brd4-dependent. Recent studies have also suggested that Brd4 recruits the P-TEFb mRNA transcription elongation factor (Cyclin T1–Cdk9 complex), which regulates mRNA transcription elongation by regulating Pol II activity [11]. Our results thus suggest that binding of Brd4 to the transcribed region of *SLC2A5* may enhance gene expression by recruiting P-TEFb and Pol II to the acetylated histones in the transcribed region, thus enhancing mRNA transcription elongation.

*SLC2A5* is induced in the small intestine of rodents during the suckling–weaning transition period when serum glucocorticoid levels increase and p44/42 MAPK is inactivated. Interestingly, we found that small-intestine expression of *Slc2a5* was significantly lower in *Brd4*^+/−^ mice compared with WT mice at 21 and 26 days after birth, suggesting that Brd4 regulates the induction of small-intestine *Slc2a5* expression during the suckling–weaning transition in mice. It should be noted that body weight was lower in *Brd4*^+/−^ mice compared with WT mice, and the lower *Slc2a5* mRNA levels in *Brd4*^+/−^ mice may thus be associated with delayed growth. However, mRNA levels of another intestinal gene, *Si*, were similar in *Brd4*^+/−^ and WT mice, implying that the decreased *Slc2a5* mRNA levels in *Brd4*^+/−^ mice were likely related to reduction of Brd4 as a result of gene targeting, rather than delayed growth. Further studies are needed to determine if acetylated histone-Brd4–P-TEFb is recruited to the transcribed region of rat jejunal *Slc2a5* during the suckling–weaning transition.

We previously demonstrated that both GR translocation from the cytosol to the nucleus, and GR binding to the promoter/enhancer region of *SLC2A5* were enhanced by co-treatment with Dex and PD in Caco-2 cells [6]. These and the current results suggest that Brd4–P-TEFb may enhance the rate of mRNA synthesis associated with triggering of the transcription initiation reaction by GR binding. Both transcription initiation and transcription elongation may thus be important for mRNA production of functional genes. However, expression of the *Si* gene during the suckling–weaning transition in mice may not be affected by Brd4 reduction via gene targeting. It is possible that the rates of expression of several genes are controlled by Brd4-associated transcription elongation and/or transcription initiation, while other genes may be controlled by Brd4. This issue should be examined in future studies.

It is still unclear which cascade promotes Brd4–P-TEFb recruitment to the transcribed region of *SLC2A5*. Because the enhancement of histone acetylation around *SLC2A5* by co-treatment with Dex and PD was closely associated with Brd4–P-TEFb binding, co-treatment may enhance histone acetylation, which may in turn promote Brd4–P-TEFb recruitment. However, the specific histone acetyltransferase (HAT) that regulates histone acetylation around *SLC2A5* has not yet been identified. GCN5 is a HAT that controls amino acid synthesis and is recruited around the transcription initiation site of intestinal genes such as *Si* and *Slc5a1* in response to a high-carbohydrate diet in rats [17]. Given that GCN5 enhances the mRNA transcriptional elongation step by acetylating histones around the transcribed region [18], it may also contribute to the induction of *SLC2A5*. A high-carbohydrate diet has also been shown to promote the association of another HAT, CREB-binding protein (CBP), with the *Mgam* gene, leading to increased *Mgam* expression in mice [19]. HATs, including GCN5 and CBP, may thus be involved in the observed regulation of *SLC2A5* expression. However, although many HATs have been identified [20], the specific HAT that is activated by Dex and PD remains unknown and should be investigated in future studies.

In conclusion, we demonstrated that Brd4 enhances the induction of *SLC2A5* in human intestinal Caco-2 cells after co-treatment with a glucocorticoid hormone agonist and a MAPK inhibitor. This induction is mediated by recruiting P-TEFb to the transcribed region and enhancing mRNA polymerase II transcription elongation. We further demonstrated that Brd4 regulated the induction of *Slc2a5* in the small intestine during the suckling–weaning transition in mice. These results suggest that Brd4–P-TEFb plays a crucial role in differentiation-associated transcription of *SLC2A5* gene in intestinal Caco-2 cells and in the small intestine of mice during weaning period.

## Author contributions

Conceived and designed the experiments: YI TG KM.

Performed the experiments and analyzed the data: YI KM.

Generated the Brd4 heterogeneous mice: AD AN KO.

Wrote the paper: YI TG TK KO KM.

## Funding

This work was supported by a Grant-in-Aid for JSPS Researcher Fellows for Young Scientists (23-9920), Grants-in-Aid for Young Scientists (22680054), a Grant-in-Aid for Scientific Research (26282023) from the Ministry of Education, Culture, Sports, Science and Technology (MEXT), and a Grant-in-Aid, and for Development of Core Technologies for Innovative Drug Development based upon IT from the Japan Agency for Medical Research and Development (AMED), and the Takeda Science Foundation.

## Conflict of interest

The authors declare that they have no conflicts of interest.

## References

[1] Suzuki T., Mochizuki K., Goda T. (2009). Localized expression of genes related to carbohydrate and lipid absorption along the crypt-villus axis of rat jejunum. Biochim Biophys. Acta.

[2] McDonald M.C., Henning S.J. (1992). Synergistic effects of thyroxine and dexamethasone on enzyme ontogeny in rat small intestine. Pediatr. Res..

[3] Koldovsky O. (1985). Response of the gastrointestinal tract to premature weaning in experimental animals. Pediatrics.

[4] Douard V., Cui X.L., Soteropoulos P., Ferraris R.P. (2008). Dexamethasone sensitizes the neonatal intestine to fructose-induction of GLUT5 transport function. Endocrinology.

[5] Gauthier R., Harnois C., Drolet J.F., Reed J.C., Vezina A., Vachon P.H. (2001). Human intestinal epithelial cell survival: differentiation state-specific control mechanisms. Am. J. Physiol. Cell Physiol..

[6] Takabe S., Mochizuki K., Goda T. (2008). De-phosphorylation of GR at Ser203 in nuclei associates with GR nuclear translocation and GLUT5 gene expression in Caco-2 cells. Arch. Biochem. Biophys..

[7] Rice J.C., Briggs S.D., Ueberheide B., Barber C.M., Shabanowitz J., Hunt D.F., Shinkai Y., Allis C.D. (2003). Histone methyltransferases direct different degrees of methylation to define distinct chromatin domains. Mol. Cell.

[8] Yan C., Boyd D.D. (2006). Histone H3 acetylation and H3 K4 methylation define distinct chromatin regions permissive for transgene expression. Mol. Cell Biol..

[9] Inamochi Y., Mochizuki K., Osaki A., Ishii T., Nakayama T., Goda T. (2010). Histone H3 methylation at lysine 4 on the SLC2A5 gene in intestinal Caco-2 cells is involved in SLC2A5 expression. Biochem. Biophys. Res. Commun..

[10] Yang X.J. (2004). Lysine acetylation and the bromodomain: a new partnership for signaling. Bioessays.

[11] Jang M.K., Mochizuki K., Zhou M., Jeong H.S., Brady J.N., Ozato K. (2005). The bromodomain protein Brd4 is a positive regulatory component of P-TEFb and stimulates RNA polymerase II-dependent transcription. Mol. Cell.

[12] Mochizuki K., Nishiyama A., Jang M.K., Dey A., Ghosh A., Tamura T., Natsume H., Yao H., Ozato K. (2008). The bromodomain protein Brd4 stimulates G1 gene transcription and promotes progression to S phase. J. Biol. Chem..

[13] Dey A., Ellenberg J., Farina A., Coleman A.E., Maruyama T., Sciortino S., Lippincott-Schwartz J., Ozato K. (2000). A bromodomain protein, MCAP, associates with mitotic chromosomes and affects G(2)-to-M transition. Mol. Cell Biol..

[14] Guo J.J., Jang R., Louder A., Cluxton R.J. (2005). Acute pancreatitis associated with different combination therapies in patients infected with human immunodeficiency virus. Pharmacotherapy.

[15] Livak K.J., Schmittgen T.D. (2001). Analysis of relative gene expression data using real-time quantitative PCR and the 2(-Delta Delta C(T)) method. Methods.

[16] Dey A., Chitsaz F., Abbasi A., Misteli T., Ozato K. (2003). The double bromodomain protein Brd4 binds to acetylated chromatin during interphase and mitosis. Proc. Natl. Acad. Sci. USA.

[17] Inoue S., Mochizuki K., Goda T. (2011). Jejunal induction of SI and SGLT1 genes in rats by high-starch/low-fat diet is associated with histone acetylation and binding of GCN5 on the genes. J. Nutr. Sci. Vitaminol..

[18] Johnsson A.E., Wright A.P. (2010). The role of specific HAT-HDAC interactions in transcriptional elongation. Cell Cycle.

[19] Mochizuki K., Honma K., Shimada M., Goda T. (2010). The regulation of jejunal induction of the maltase-glucoamylase gene by a high-starch/low-fat diet in mice. Mol. Nutr. Food Res..

[20] Marmorstein R., Roth S.Y. (2001). Histone acetyltransferases: function, structure, and catalysis. Curr. Opin. Genet. Dev..

